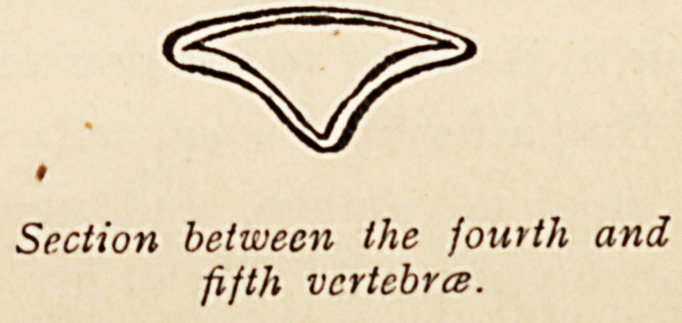# Some Remarks on Spinal Anæsthesia as Based upon the Personal Observation of Thirty Cases

**Published:** 1907-12

**Authors:** Ernest W. Hey Groves

**Affiliations:** Assistant-Surgeon, Bristol General Hospital.


### SOME REMARKS ON SPINAL ANÆSTHESIA, AS BASED UPON THE PERSONAL OBSERVATION OF THIRTY CASES.

BY

ERNEST W. HEY GROVES, M.S. Lond., F.R.C.S.

*Assistant-Surgeon, Bristol General Hospital.*

So much has recently been said and written on this subject, that I do not propose to attempt any review of the history and literature, but merely to record certain points of interest and difficulty that have occurred in actual practice. It is only by the noting of difficulties and drawbacks of any new method that these may be met and eventually overcome. The operations referred to are the following:—

| | | | |
|---|---|---|---|
| Colotomy .. .. .. .. | 2 | Bursæ .. .. .. .. | 2 |
| Hernia .. .. .. .. | 7 | Bone necrosis .. .. .. | 3 |
| Intestinal obstruction | 1 | Exostosis .. .. .. .. | 1 |
| Exploration of bladder | 1 | Hammer toe .. .. .. | 1 |
| Extravasation of urine | 1 | Amputation of toe .. | 1 |
| Amputation of hip .. | 2 | Varicose veins .. .. .. | 4 |
| Hydrocele .. .. .. .. | 3 | Disease of ankle joint | 1 |

The earliest of these operations was performed on September 9th, 1904, and was for an acute suppuration round the knee joint, in a girl of 18; $\frac{1}{4}$ gr. cocaine was used and the result was very good. For the next ten cases the same method was followed. But it soon became evident that the method had great disadvantages, viz. grave danger during the anæsthesia and severe reaction afterwards, and these led to the abandoning of cocaine for stovaine, which was the agent used in all the rest of the cases. The cocaine cases were none of them of a serious character, and two instances will suffice to illustrate the drawbacks referred to.

J. W., man, aged 47. Radical operation for hydrocele. $\frac{1}{5}$ gr. cocaine. Complete analgesia below navel; pulse fell to 72, and became very irregular; the face became ashy pale; patient said he felt desperately ill. Soon revived after ½ oz. brandy, but had severe vomiting and headache afterwards.

H. C., man, aged 29. Operation for removing a sequestrum from the femur. ¼ gr. cocaine. Directly after the operation his temperature rose to 104° F. with a slight rigor. This was followed by vomiting and a severe pain in the head and back, which lasted about twenty-four hours.

These, I believe, are fairly typical of the troubles which occur during and after spinal cocainisation. In its toxic effects the drug, when used as a spinal anæsthetic, causes a slowing and irregularity of the pulse which may end fatally. And in its lesser degrees this phenomenon cannot be very rare, as I experienced it twice in eleven cases. The severe headache, backache and sharp rise of temperature with vomiting are quite as or even more severe than the after effects of a general anæsthetic, and these symptoms occur very frequently.

But the era of cocaine for spinal anæsthesia is now past, and its derivatives—stovaine, novococaine and tropacocaine—are on their trial. And it may, I think, be definitely stated that stovaine, at any rate, has been proved to avoid the dangers of cocaine, and the other drugs are even more highly spoken of, but they do not enter into the present series.

The first point to be raised is the certainty and completeness of the anæsthesia. Now this seems to depend chiefly on the possibility of injecting the fluid into the spinal sub-dural space, and this in its turn is indicated by obtaining a free flow of cerebro-spinal fluid from the canula before injection. If the fluid can be obtained freely, then the occurrence of complete analgesia after an appropriate dose of the stovaine has been injected is certain. In four of my cases the fluid could not be obtained from the spine, and in all the anæsthesia was a failure, and in most published series there have been at least 3 per cent. of failures from the same cause. And if this cannot be remedied it constitutes a very great drawback, inasmuch as one can never be so certain of the spinal anæsthesia as to be able to dispense with an anæsthetist in cases of emergency which occur at a distance from help. And it is therefore worth while to consider the causes of this failure, in order to see whether it can be avoided. If the vertebral column be cut across between the fourth and fifth lumbar vertebræ, and again between the second and third, the following appearances are noted inside the vertebral canal. In the former case the canal consists of a triangle about $\frac{3}{4}$ in. across and $\frac{1}{4}$ in. depth, whose basal angles are very narrow. The dura is, however, closely attached to the bone. In the latter case the canal is 1 in. wide and over $\frac{1}{2}$ in. deep, with much more widely open angles. But here the dura is hardly attached to the bone at all, and if, therefore, there is not much tension in the sub-dural space, this

*Section of the vertebral canal and dura between the second and third lumbar vertabræ.*

*Section between the fourth and fifth vertebræ.*

space can easily be obliterated by the collapse of its walls. It is evident from these considerations that two causes may prevent a puncturing needle from withdrawing spinal fluid. First, the needle may not get into the vertebral canal at all, which is especially liable to occur in the lowest space; and, secondly, the needle may push the membranes in front of it, and then merely transfix the two layers of collapsed spinal theca. This is more likely to be the case in one of the upper lumbar intervals. I have found that the cases of failure have all been anæmic or old feeble people, in whom the tension of the spinal fluid is probably low. And in them also the ventricles of the brain are large, and the communication with the sub-dural space small, all of which factors would lessen the likelihood of the spinal fluid escaping from a puncture.

If these suggestions are correct, the following precautions must tend to lessen the chances of failure. First thrust the needle into the space between the fourth and fifth lumbar spines, this being indicated by the point midway between the highest points of the iliac crests. The needle should be very sharp and four inches long. It must be kept accurately in the mid-line, and follow strictly the sagittal plane. If this fails, it is much better to try the space between the second and third vertebræ than to make repeated attempts in the same space. It should if possible, be done in the sitting or standing position with the head bent forward. The use of a needle provided with a trocar, and also with a lateral as well as a terminal opening, is to be recommended

As regards the dose required, in most cases ½ c.c. of solution (1 mg. of stovaine) is sufficient; but the susceptibility of patients varies very much, and this dose will produce a prolonged total and extensive analgesia in one patient and only a short analgesia in another. So that in cases of abdominal disease, or those in which the operation is likely to last more than one hour, it is better to use double the above dose.

But, after all, the capacity of the measure to prevent shock is its chief advantage, and upon this its use in the future will probably depend. Six of my cases illustrate this point.

T. J., man, aged 52. Old case of traumatic stricture of the urethra. High grade of retention of urine for ten days with signs of renal sepsis. Extensive extravasation of urine, the parts being gangrenous and emphysematous. Pulse 140; temperature sub-normal. 1 c.c. stovaine injection. Multiple incisions and opening through the stricture into the bladder. General condition was unchanged during the operation, but he died about three hours later.

G. S., aged 63. Old inguinal hernia. Became strangulated nearly a week before admission to the Cossham Hospital. General condition bad; small, irregular pulse; some vomiting; abdomen distended; right inguinal hernia. At 4 p.m. gave 1 c.c. stovaine and operated. Six inches of gangrenous small intestine resected, but peritonitis evidently already existed, and he died six and a half hours later. The pulse fell to 108, and he seemed actually relieved during the operation.

J. K., man, aged 60. Acute spreading emphysematous gangrene of the right leg. 1 c.c. stovaine. Amputation through the hip-joint. Pulse and general condition was better directly after the operation, but he died three hours after.

A. G., boy, aged 19. Huge sarcoma of the adductor muscles of the right thigh. ½ c.c. stovaine. Amputation through the hip by the anterior racket method The pulse and blood pressure were noted every ten minutes before, during and after the operation. The pulse dropped from 140 to 108, and the blood pressure from

115 m.m. to 70 m.m., and then rose to 74 m.m. He was bright, cheerful and constantly talking to the nurse. He complained once of feeling faint, but was better after a drink of water. A few hours after the operation he was bright and cheerful; his pulse remained at 108, but was rather small and compressible.

M. G., woman, aged 65. Had had colotomy performed eight months previously for cancer of the rectum. Intestinal obstruction had again occurred, and her abdomen was hugely distended with visible peristalsis. $\frac{1}{2}$ c.c. stovaine. Median incision. Intestines matted together in the pelvis. Paul's tube tied into the small intestine. Died about twenty-four hours later.

E. A., aged 55. Strangulated right inguinal hernia; fæcal vomiting, great distension, and very poor general condition, the obstruction having lasted several days. $\frac{1}{2}$ c.c. stovaine. A double loop of gut was strangulated, but not gangrenous; it was easily reduced. A very copious fluid motion, amounting to several pints, occurred on the table at the moment of reduction, and he became faint and collapsed. The whole operation only lasted twenty minutes, but he died an hour later.

These six cases represent, of course, the most desperate that can ever come under the surgeon's care, but for which an attempt must be made to save life. In four there can be no doubt that death would either have occurred on the table or without the return of consciousness. Spinal anæsthesia in such cases is of the utmost value. It almost entirely abolishes nerve shock, though of course it cannot prevent the effects of hemorrhage. It allows the patient full retention of his consciousness, and he can, at any rate, be safely returned to bed and see and speak with his relatives. And if it is possible for an operation in such desperate cases to succeed, it will have a far better chance than with general anæsthesia. With regard to the last case, I confess I am in great doubt. Other observers have noted the power of stovaine to produce intestinal contraction, and it would appear that in this instance the sudden evacuation of the bowels brought about the fatal collapse.

Then there are certain cases in which the presence of some other disease makes a general anæsthetic very dangerous. Such are particularly, severe lung diseases and diabetes. One example will illustrate this.

A. M., boy, aged 14. A congenital syphilitic, had double pneumonia, and an acute periostitis (probably pneumococcal)

of the left femur. Under ½ c.c. stovaine, the femur was cut down upon, and the bare bone exposed and drained. This had no ill-effect on his general condition, except that he had some retention of urine afterwards. He died from the pneumonia a week later.

As regards any ill-effects in the after history following stovaine anæsthesia, these are very rare. Headache, backache, vomiting or rise of temperature occur very seldom, and are almost negligible. In one instance, that of a man aged 52 with caries of the ankle-joint, an acute bed-sore developed three days later over the sacrum, and may have been due to some trophic effect of the spinal analgesia.

In conclusion, I would submit that stovaine-spinal-anæsthesia presents in ordinary uncomplicated cases no special advantage over general anæsthesia, and on the contrary has the draw-backs of an uncertainty of attainment in about 4 per cent. of cases, and of not allowing the actual dose to be graduated to suit the idiosyncrasies of the patient. But it is valuable under the following conditions in operations on the lower abdomen or legs :—

(1) Operations involving the danger of great shock. (2) Operations performed in conditions of desperation, where it is doubtful that the patient will leave the table alive. (3) In severe diabetes, acetonuria or acid intoxication, severe lung diseases. (4) In emergency cases, *e.g.* a compound fracture or strangulated hernia, where an anæsthetist is unavailable.

## Figures and Tables

**Figure f1:**
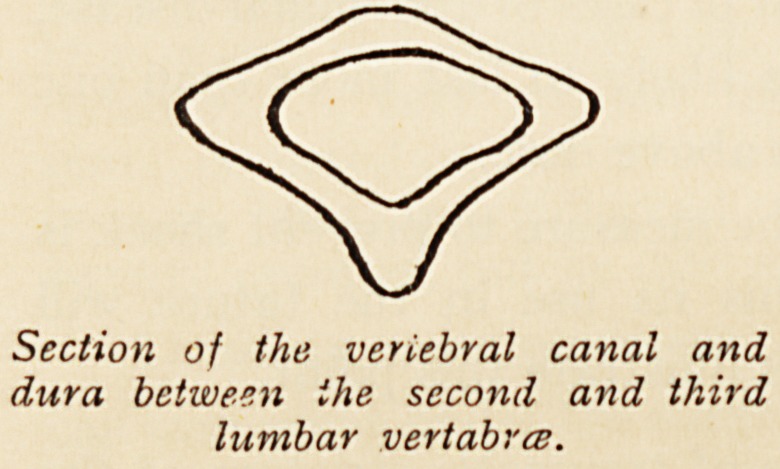


**Figure f2:**